# Modeling the Electrical Activity of the Heart via Transfer Functions and Genetic Algorithms

**DOI:** 10.3390/biomimetics9050300

**Published:** 2024-05-18

**Authors:** Omar Rodríguez-Abreo, Mayra Cruz-Fernandez, Carlos Fuentes-Silva, Mario A. Quiroz-Juárez, José L. Aragón

**Affiliations:** 1 Centro de Física Aplicada y Tecnología Avanzada, Universidad Nacional Autónoma de México, Santiago de Querétaro 76230, Mexico; omar.rodriguez@upq.edu.mx; 2 Division de Tecnologías Industriales, Universidad Politécnica de Querétaro, Santiago de Querétaro 76240, Mexicocarlos.fuentes@upq.mx (C.F.-S.)

**Keywords:** modeling, ECG, transfer function, genetic algorithm, metaheuristic optimization

## Abstract

Although healthcare and medical technology have advanced significantly over the past few decades, heart disease continues to be a major cause of mortality globally. Electrocardiography (ECG) is one of the most widely used tools for the detection of heart diseases. This study presents a mathematical model based on transfer functions that allows for the exploration and optimization of heart dynamics in Laplace space using a genetic algorithm (GA). The transfer function parameters were fine-tuned using the GA, with clinical ECG records serving as reference signals. The proposed model, which is based on polynomials and delays, approximates a real ECG with a root-mean-square error of 4.7% and an $R^{2}$ value of 0.72. The model achieves the periodic nature of an ECG signal by using a single periodic impulse input. Its simplicity makes it possible to adjust waveform parameters with a predetermined understanding of their effects, which can be used to generate both arrhythmic patterns and healthy signals. This is a notable advantage over other models that are burdened by a large number of differential equations and many parameters.

## 1. Introduction

Mathematical models have become indispensable across many disciplines for predicting the dynamic behaviors of complex systems at different scales. Generally, these models are described by a set of differential equations that represent the variation in the system with respect to certain independent variables, typically time. Recently, there has been a notable advancement in the precision of mathematical models, particularly in the realm of biological phenomena. In the last century, these advancements were unattainable because of computational resource constraints. This progress has enabled the simulation of intricate biological processes with unprecedented accuracy, thereby revolutionizing our ability to understand and manipulate biological systems [1].

The human heart is one of the most intriguing biological systems and studied from various perspectives. The complexity inherent in this analysis demands the integration of biological, medical, and engineering disciplines to understand the dynamics of cardiac function. Many efforts have been made to understand the characteristics of the heart’s function, from its physiology [2] to its interactions with various systems and organs of the body, including the brain [3]. However, there are still many open questions that need to be answered. One such question revolves around the mechanisms underlying certain cardiac pathologies such as arrhythmias or heart failure. Importantly, diseases associated with the heart are considered primary causes of death worldwide [4]. Addressing investigations along this line not only holds the promise of improving diagnostic and therapeutic approaches but also deepens our fundamental understanding of cardiac function and paves the way for more effective interventions and treatments of these diseases in the future.

Although complete cardiac studies can be complicated or expensive, various experimental methods allow heart analysis, such as electrophysiological studies and optical imaging of the electrical activity of the heart. Electrocardiograms (ECG) are the most common analysis method [5], through which a wide variety of heart diseases can be detected in a non-invasive manner. ECG signals are captured through electrodes strategically placed on various parts of the body. These electrodes collect electrical information from different angles, leading to so-called “leads” that offer different perspectives on the heart’s electrical activity. These signals reflect transmembrane ionic currents in the heart [6]. Typically, a healthy heartbeat manifests in two phases: systole, corresponding to contraction, and diastole, which is indicative of relaxation. Given the accessibility and minimal risk to patients, coupled with the valuable information it provides, ECGs remain a cornerstone tool for diagnosing heart conditions [7,8].

As previously mentioned, comprehending electrical activity behavior from a mathematical standpoint is crucial for diagnosing heart conditions [9]. To this end, various mathematical models have been proposed [10] to detect arrhythmias or other pathologies, comprehend the defibrillation process, and analyze the impact of electrical disturbances on the heart [11]. Remarkably, incorporating all variables affecting the electrical activity of the heart results in a complex model that is impractical for real-world applications. Furthermore, certain variables or parameters of the model may be challenging or impossible to measure. Examples of these models include the bidomain model and the EMI model, which takes its name from the extracellular space (E), cell membrane (M), and intracellular space (I) [12]. Although these models offer high precision, they incur substantial computational costs [13]. Other research efforts have focused on proposing mathematical models to reproduce the electrical activity of the heart at a macroscopic level through ECG signals, allowing a balance between simplicity and precision [14,15]. The purpose of modeling is to provide simplified representations of complex phenomena. Each model adapts differently to the real characteristics of a phenomenon. A novel model of the heart’s electrical activity can offer fresh insights into its functioning. Regarding clinical use, the construction of a new model is an alternative that allows for generating a comparative database of synthetic ECG signals used to train arrhythmia and heart disease detection systems and make them more robust.

On the other hand, research on artificial intelligence (AI), including evolutionary computation, has witnessed exponential growth in recent years, largely attributed to a remarkable surge in computing power. This has facilitated the resolution of problems across numerous scientific domains that lack exact solution methods or are computationally infeasible owing to their complexities. Currently, there is a growing trend in the use of AI tools to diagnose cardiac issues by analyzing ECGs [7]. However, AI can also be used to assist in creating models that more accurately represent the dynamics of heartbeats through metaheuristic algorithms. An illustrative example is presented in [16], where a mathematical model was developed using metaheuristic algorithms.

In this study, we introduce a dynamical model, grounded in transfer functions [17,18], which describe the heart’s electrical activity at a macroscopic level. Our model comprises three low-order transfer functions coupled with time delays whose parameters are obtained through metaheuristic algorithms, specifically genetic algorithms (GA). Using a database of real electrocardiograms as reference signals, our model can be tailored to various ECG waveforms, a novel approach that has not been previously explored in the literature to our knowledge. Through minimization of the integral square error, the GA determines the parameter range for healthy ECGs, as well as for ECGs exhibiting arrhythmias. The results demonstrated that our model effectively captured real ECG signals, with a root mean square error (RMSE) of 4.7% for healthy signals and an average RMSE of 7.2% for arrhythmia signals. Finally, we compared our model with existing proposals, highlighting its distinctiveness in allowing each electrocardiogram wave to be independently adjusted, with parameters obtained through evolutionary computation rather than manual tuning. Thus, our study underscores the utility of AI-guided transfer function modeling as a practical and valuable tool for investigating ECG signals.

## 2. Background and Related Works

**Figure 1:**
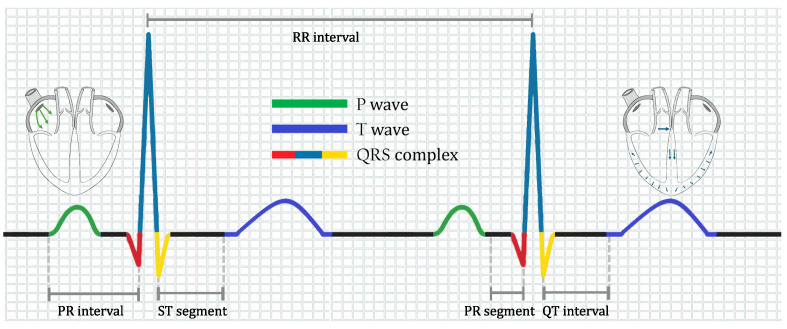
Ideal ECG for a healthy heart.

An ECG is a recording of the electrical activity of the heart through electrodes placed on the skin. These electrodes collect the voltage changes caused by the depolarization and repolarization processes that occur with every heartbeat. The peaks and valleys of the ECG signal are labeled as the P wave, QRS complex, and T wave, as shown in Figure 1.

As depicted in Figure 1, an ECG is composed of segments, intervals, and waves. The P wave originates from the depolarization of the atria. In contrast, the QRS complex originates from contraction of the lower chambers of the heart (depolarization of the ventricles). The T-wave originates from the repolarization of the ventricles. The PR segment moves from the beginning of the P-wave to the beginning of the QRS complex, representing conduction through the atria. Finally, the QT interval extends from the start of the QRS complex to the final T wave, containing information on the processes of ventricular depolarization and repolarization. We refer the reader to ref. [19] for a detailed explanation.

Several models have been developed to represent electrical cardiac dynamics. The best-known model is the bidomain model, which is a set of mathematical equations that describe the electrical properties of cardiac tissue [20]. This model is widely used when maximum precision is required in numerical simulations and the computational cost is not important. However, mathematical modeling requires a balance between detail and tractability; thus, more manageable models have been proposed, which are briefly discussed below.

### 2.1. Ring of Three-Coupled Oscillators

One of the first ideas for obtaining simplified models of the heart was to consider a set of nonlinear oscillators. A notable example is a model consisting of a ring of three oscillators coupled with delays, which was developed by employing a set of six ordinary differential equations. In this model, each oscillator corresponded to a natural pacemaker in the heart [21]. The equations governing this model are as follows:

(1) $$\begin{array}{cc} {\dot{x}}_{1}= & x_{2},\\ {\dot{x}}_{2}= & -a_{SA}x_{2}\left(x_{1}-\omega_{SA_{1}}\right)\left(x_{1}-\omega_{SA_{2}}\right)+\rho_{SA}\sin\left(\omega_{SA}t\right)-x_{1}\left(x_{1}-d_{SA}\right)\left(x_{1}-e_{SA}\right)\\ \; & -K_{SA-AV}\left(x_{1}-x_{3}^{\tau_{SA-AV}}\right)-k_{SA-HP}\left(x_{1}-x_{5}^{\tau_{SA-AV}}\right),\\ {\dot{x}}_{3}= & x_{4},\\ {\dot{x}}_{4}= & -a_{VA}x_{4}\left(x_{3}-\omega_{AV_{1}}\right)\left(x_{3}-\omega_{AV_{2}}\right)+\rho_{AV}\sin\left(\omega_{AV}t\right)-x_{3}\left(x_{3}-d_{AV}\right)\left(x_{1}-e_{AV}\right)\\ \; & -K_{AV-SA}\left(x_{3}-x_{1}^{\tau_{AV-SA}}\right)-k_{AV-HP}\left(x_{3}-x_{1}^{\tau_{SA-AV}}\right),\\ {\dot{x}}_{5}= & x_{6},\\ {\dot{x}}_{6}= & -a_{HP}x_{6}\left(x_{5}-\omega_{HP_{1}}\right)\left(x_{5}-\omega_{HP_{2}}\right)+\rho_{HP}\sin\left(\omega_{HP}t\right)-x_{5}\left(x_{5}-d_{HP}\right)\left(x_{5}-e_{HP}\right)\\ \; & -K_{HP-SA}\left(x_{5}-x_{1}^{\tau_{HP-SA}}\right)-k_{HP-AV}\left(x_{3}-x_{3}^{\tau_{SA-AV}}\right), \end{array}$$

where $a_{node},\omega_{node_{i}},d_{node}$, and $e_{node}$ are parameters of the system, and node=SA,AV,HP, stand for sinoatrial, atrioventricular, and His–Purkinje nodes, respectively. τ is the time delay, $\rho_{node}$ is the amplitude of the external stimulus, and $\omega_{node}$ is its frequency. Finally, the ECG signal can be determined by

(2) $$ECG\left(t\right)=\left(\alpha_{0}+\alpha_{1}x_{1}+\alpha_{3}x_{3}+\alpha_{5}x_{5}\right)\beta_{G},$$

where $\alpha_{i}$, i=1,2,3, are parameters weighting each system variable and $\beta_{G}$ is used as a global scaling factor.

### 2.2. Heterogeneous Nonlinear Oscillators

Heterogeneous nonlinear oscillators consist of modified Van Der Pol and FitzHugh–Nagumo oscillators, which capture the action potentials of primary natural pacemakers and the electrical responses of cardiac muscle tissues (atrial and ventricular muscles) [22]. Subsequently, this model was adapted to represent ventricular fibrillation as an instance of chaos [23]. The equations governing natural pacemakers in this model are as follows:

(3) $$\begin{array}{c} {\dot{x}}_{i}=y_{i},\\ {\dot{y}}_{i}=-a_{i}y_{i}\left(x_{1}-u_{i}\right)-f_{i}x_{i}\left(x_{i}+e_{i}\right)+K_{node}\left(y_{i-1}^{\tau_{node}}-y_{i}\right). \end{array}$$

The sinoatrial node corresponds to i=1, the atrioventricular node corresponds to i=2, and the His–Purkinje term corresponds to i=3. Coefficients $a_{i},f_{i},u_{i},d_{i}$, and $e_{i}$ are the parameters of each oscillator. Finally, a complete description of the ECG signal is given by

(4) $$ECG\left(t\right)=z_{0}+\alpha_{1}z_{1}-\alpha_{2}z_{2}+\alpha_{3}z_{3}+\alpha_{4}z_{4},$$

where $z_{0}$ adjusts the baseline of the ECG signal and $z_{i}$ corresponds to the depolarization and repolarization processes represented by the P-wave (i=1), Ta-wave (i=2), QRS complex (i=3), and T-wave (j=4), which are defined as follows:

(5) $$\begin{array}{c} {\dot{z}}_{j}=k_{j}\left(-c_{j}z_{j}\left(z_{j}-\omega_{j1}\right)\left(z_{j}-\omega_{j2}\right)-b_{j}v_{j}-g_{j}v_{j}z_{j}+I_{j}\right),\\ {\dot{v}}_{j}=k_{j}h_{j}\left(z_{j}-v_{j}\right), \end{array}$$

where $k_{j}$ is a scaling factor; $c_{j}$ governs the amplitude of the pulse; $b_{j}$ and $g_{j}$ modify the rest state; and $h_{j}$, $\omega_{j1}$, and $\omega_{j2}$ control the duration of the action potential, the excitation threshold, and excited state of each oscillator, respectively. $I_{j}$ is the magnitude of the stimulation current that couples the natural pacemakers to the cardiac muscles.

### 2.3. Reaction–Diffusion Model

The Reaction–diffusion spatially discretized model is a set of three nonlinear oscillators obtained from the spatial discretization of a reaction-diffusion model [24]. This model generates normal ECGs and cardiac arrhythmias. The set of ordinary differential equations that describe the model is as follows:

(6) $$\begin{array}{cc} {\dot{x}}_{1} & =x_{1}-x_{2}-Cx_{1}x_{2}-x_{1}x_{2}^{2},\\ {\dot{x}}_{2} & =-Hx_{1}-3x_{2}+Cx_{1}x_{2}+x_{1}x_{2}^{2}+\beta \left(x_{4}-x_{2}\right),\\ {\dot{x}}_{3} & =x_{3}-x_{4}-Cx_{3}x_{4}-x_{3}x_{4}^{2},\\ {\dot{x}}_{4} & =Hx_{3}-3x_{4}-Cx_{3}x_{4}-x_{3}x_{4}^{2}+2\beta \left(x_{2}-x_{4}\right), \end{array}$$

where β is the coupling parameter between the oscillators, and H and C are parameters that control the dynamics of the system. The ECG signal is obtained using the following linear combination of variables:

(7) $$ECG\left(t\right)=\alpha_{1}x_{1}+\alpha_{2}x_{2}+\alpha_{3}x_{3}+\alpha_{4}x_{4},$$

where the parameters $\alpha_{i}$ and i=1,2,3 are the weights.

### 2.4. Extended Dynamical Model Based on a Quasi-Periodic Motion

Originally developed in ref. [25], this model consists of a set of three coupled ordinary equations that can generate simple ECG signals for both normal heartbeats and arrhythmias, with simple parameter variations. The original equations for this model are as follows:

(8) $$\begin{array}{cc} \dot{x} & =\alpha x-\omega y,\\ \dot{y} & =\alpha y-\omega x,\\ \dot{z} & =\sum_{i\in (P,Q,R,S,T)}a_{i}\Delta \theta_{i}\exp-\frac{\Delta \theta_{i}^{2}}{2b_{i}^{2}}-\left(z-z_{0}\right), \end{array}$$

where $\alpha =1-\sqrt{x^{2}+y^{2}}$, $\Delta \theta_{i}=\left(\theta -\theta_{i}\right)$ mod 2π, θ=arctan(y,x), and ω is the angular velocity. $z_{0}=A\sin\left(2\pi f_{2}t\right)$ is the baseline, where *A* is the amplitude and $f_{2}$ represents the respiratory frequency. Here, the *z* variable yields a synthetic ECG waveform.

Subsequently, a Gaussian wavelet-based state space was developed using (8). In this modified model, characteristic waves, such as atrial and ventricular complexes, can be controlled individually [26], and it was shown that this model can accurately represent a human heartbeat. The modified model is described by the equations

(9) $$\begin{array}{cc} \dot{x} & =\alpha x-\omega y,\\ \dot{y} & =\alpha y-\omega x,\\ \dot{P} & =\sum_{i\epsilon (P^{-},P^{+})}a_{i}\Delta \theta_{i}\exp-\frac{\Delta \theta_{i}^{2}}{2b_{i}^{2}}-\left(P-P_{0}\right),\\ \dot{C} & =\sum_{i\epsilon (Q,R,S)}a_{i}\Delta \theta_{i}\exp-\frac{\Delta \theta_{i}^{2}}{2b_{i}^{2}}-\left(C-C_{0}\right),\\ \dot{T} & =\sum_{i\epsilon (T^{-},T^{+})}a_{i}\Delta \theta_{i}\exp-\frac{\Delta \theta_{i}^{2}}{2b_{i}^{2}}-\left(T-T_{0}\right). \end{array}$$

Finally, the synthetic ECG is obtained as

(10) ECG(t)=P(t)+C(t)+T(t).

### 2.5. Periodically Kicked Network of RLC Oscillators

This linear model is based on two RLC linear oscillators excited by a pulse signal train, including a time delay to account for the delay in the electrical transport from the atria to the ventricles [27]. This model has the advantage that analytical solutions can be obtained when a single impulse is applied. A critical feature of this model is that each parameter modifies a specific waveform characteristic of the synthetic ECG signals produced. Although this model has some limitations, it can show that the main signals of an ECG can be reproduced using linear models. The equation describing the ECG is as follows:

(11) $$\begin{array}{cc} ECG(t)= & 2A\gamma \omega_{0}^{2}\left(\frac{e^{-}\frac{\alpha +\sqrt{\alpha^{2}-4\omega_{0}^{2}}}{2}u\left(t-\tau \right)\left(e^{t-\tau \sqrt{\alpha^{2}-4\omega_{0}^{2}}}-1\right)u\left(t-\tau \right)}{\sqrt{\alpha^{2}-4\omega_{0}^{2}}}\right.\\ \; & \left.+\frac{e^{-\frac{\alpha }{2}}\sinh\left(\frac{\sqrt{\alpha^{2}-4\omega_{0}^{2}}}{2}t\right)}{\sqrt{\alpha^{2}-4\omega_{0}^{2}}}-\frac{e^{\left(-\frac{\alpha }{2(1+C_{X}\omega_{0})}\right)t}\sinh\left(\frac{\sqrt{\alpha^{2}-4\omega_{0}^{2}\left(1+C_{X}\omega_{0}\right)}}{2(1+C_{X}\omega_{0})}t\right)}{\sqrt{\alpha^{2}-4\omega_{0}^{2}\left(1+C_{X}\omega_{0}\right)}}\right), \end{array}$$

where $\alpha =\frac{1}{RC}$ denotes the damping factor, $\omega_{0}=\sqrt{\frac{1}{LC}}$ denotes the natural frequency, and γ denotes the input pulse width. *R*, *C*, and *L* denote the resistance, capacitance, and inductance, respectively.

## 3. Materials and Methods

Here, we will show that a set of three second-order transfer functions (one function per repolarization and depolarization process) can generate waveforms that resemble clinical ECGs. The model is stimulated by a cyclical impulse signal.

Second-order transfer functions have two basic polynomial representations: standard and non-standard forms. In this study, the non-standard form was used, since investigations like [28] explained that if the variations of a phenomenon can be measured or predicted theoretically, then they can be represented by a non-standard transfer function. Additionally, non-standard transfer functions allow the use of proper transfer functions; that is, with a polynomial in the numerator a degree lower than the polynomial of the numerator. Therefore, non-standard transfer functions can present much more complex dynamics than standard second-order transfer functions, adding parameters such as the coefficient of the zero in the numerator, which is not considered within a standard function. This explanation was added in Section 3. Subsequently, we use time delays to couple the different transfer functions. The model for reproducing a heartbeat (HB) is described by the sum of the three transfer functions, as follows:

(12) $$HB\left(s\right)=\sum_{i=1}^{3}k_{i}e^{-r_{i}s}\frac{a_{i}s-b_{i}}{s^{2}+c_{i}s+d_{i}},$$

where the transfer functions corresponding to i=1, 2, and 3 represent the P-wave, the QRS complex, and the T-wave, respectively. a,b,c,d,r, and *k* are the model parameters. In particular, *k* is a gain that allows controlling the response amplitude, *a* represents the position of the zero in the transfer function, *r* is the time delay associated with the coupling of every signal, *c* is a parameter that allows controlling the damping factor, and *d* and *b* are related to the natural frequency of the system.

Equation (12) is driven by a periodic pulse train, which represents the rhythmic nature of heartbeats. Hence, the entire ECG output can be defined as

(13) $$ECG\left(s\right)=\sum_{i=1}^{3}\frac{k_{i}e^{-r_{i}s}}{1-e^{\frac{s}{f}}}\frac{a_{i}s-b_{i}}{s^{2}+c_{i}s+d_{i}},$$

where *f* denotes the pulse train frequency. The next task is to find the parameter values for (12) and (13) to reproduce the particular synthetic ECG waveforms. Given the complexity of estimating multiple coefficients, metaheuristic algorithms have emerged as plausible choices. These algorithms exhibit versatility in addressing various problems across different domains, as evidenced by their widespread application [29]. Metaheuristic algorithms have the advantage of easily adapting to any problem and running in parallel when the processing time increases. However, it is essential to note that metaheuristic algorithms do not guarantee a global optimum, implying that they may not always converge to the best solution. Nevertheless, by appropriately configuring the algorithm, solutions that satisfy the constraints of the problem can be obtained.

Genetic algorithms (GA) stand out as one of the most widely used and well-known metaheuristic algorithms [30]. Inspired by natural selection, a GA mimics the process of selecting the most advantageous genes for inheritance by successive generations. The various versions of this algorithm can yield different results. Nonetheless, a fundamental phase common to most GA iterations is selection, in which a fitness function assesses all potential initial random solutions to identify the best-performing ones. Mutation introduces random variations in the solutions to prevent convergence to local maxima, whereas crossover facilitates the combination of two strong solutions.

We used a GA to explore the parameters of (12) and (13) to generate an ECG signal that closely resembles a real signal. Algorithm 1 is the pseudocode describing the GA utilized in this study. Note that elitism was employed to ensure descent of the most suitable genes.
**Algorithm 1** Pseudocode for the used GA1:**Begin**2:**Select hyperparameters**3:Set the initial population P; each individual is a vector with random coefficients corresponding to Equation (12).4:**while** fitness function ≤ 30,000 **do**5:    Calculate fitness of each individual of P, defined by the Integral Square Error.6:    Selection of P members with lower fitness value, according to biological pressure.7:    Crossover parents with lowest fitness, using a random single point to create the union.8:    Random change of a value in individuals (mutation).9:    Generate a new generation of individuals P using elitism and the members with the lowest fitness.10:**end while**11:Keep the best solution (minimum fitness)12:**end**

The GA starts with a random population and assigns a "fitness" value to each random solution. A fitness function is selected to define the performance of each solution, with the most common being the mean square error (MSE), root mean square error (RMSE), and integral square error (ISE). For this study, the ISE was used as the fitness function, as follows:

(14) $$ISE=\int \varepsilon {\left(t\right)}^{2}dt,$$

where ε is defined as

(15) $$\varepsilon \left(t\right)=ECG_{real}\left(t\right)-ECG_{simulated}\left(t\right)$$

The selection was carried out using a tournament of size 3, and the remaining parameters of the GA are summarized in Table 1.

Once the fitness function and parameter values of the GA are defined, the parameter values of the proposed model itself must be fine-tuned using clinical ECG registers as reference waveforms. In this study, the publicly available database PhysioNet [31,32] was used. This extensively used database grants access to 12-lead ECGs and encompasses 310 electrocardiogram records from 90 individuals, comprising 46 women and 44 men aged between 13 and 75 years. The database comprises two channels: the first channel contains RAW data, and the second channel contains filtered data. Each ECG recorded in this database has a resolution of 12 bits and a range error rate of ±10 mV. Labeled as patients with normal ECGs, each ECG signal was stored for 20 s and digitized at a sample rate of 500 Hz. This database was selected owing to its widespread recognition, extensive acquisition of details, and comprehensive nature.

The heartbeats from each individual in the study were manually segmented using the filtered channel. The system exhibited improved performance with noise-free signals. Nonetheless, this enabled the assessment of the method’s efficacy against a complex database encompassing variations that can be expected in real ECGs. Upon segmentation of the data, one of the 90 patients selected from the database was randomly chosen to initiate the model parameterization. Subsequently, the range of each parameter was defined so that it could be better adjusted to the database.

After completing the process for a single patient, the results were replicated for the remaining 89 patients, to determine the range of variation for each parameter. The simulations were conducted in a MATLAB-Simulink environment. MATLAB utilizes a genetic algorithm to produce coefficients assessed in the transfer functions built in Simulink. Subsequently, Simulink returned the simulated ECG signals, and MATLAB continued to evaluate the fitness using the remainder of the genetic algorithm. The model was solved using a fixed step of 0.0001 s and an automatic solver selection. The simulation times were selected based on the ECG signals used. All simulations were conducted on a computer with the following specifications: an 11th generation Intel(R) Core(TM) i7-1165G7 processor running at 2.8 GHZ, with 16 GB of RAM.

## 4. Results and Discussion

**Figure 2:**
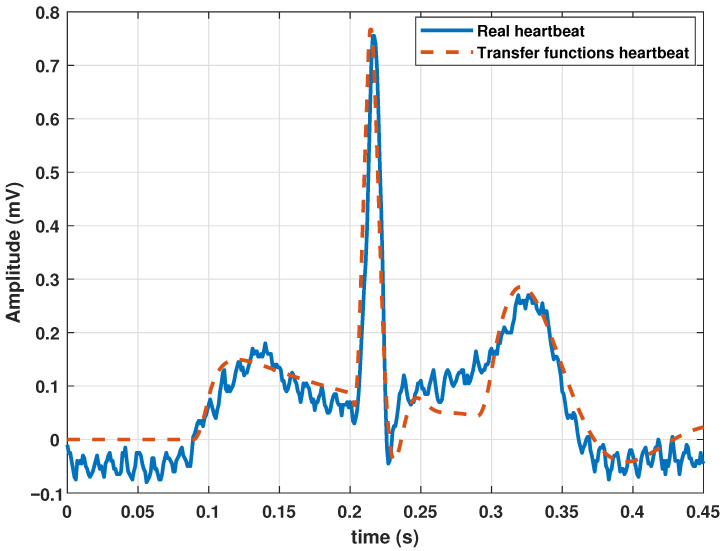
Comparison between a heartbeat obtained from the database and a heartbeat generated by the transfer function Model (13) tuned by a genetic algorithm.

To synthesize a single heartbeat, the model parameters were adjusted by executing the GA (Algorithm 1 and the parameters of Table 1), using a clinical heartbeat as a reference waveform. As mentioned above, a real heartbeat from a randomly selected patient in the database (randomly selected) was used. The numerical results of the adjustment are presented in Table 2, and the resulting signal is shown in Figure 2.

**Figure 3:**
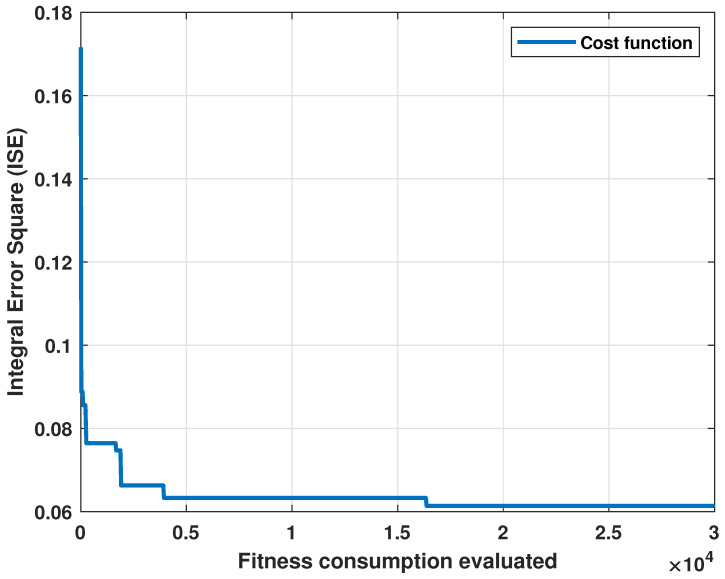
Cost evolution for each evaluation of the fitness function.

The cost graph per iteration is shown in Figure 3. Standard statistical metrics were used to analyze the behavior of the method. The statistical results for this model compared to the actual heartbeat had an RMSE of 0.0471, $R^{2}$ of 0.7153, and an MBE of −0.0204.

These results show that the proposed model could represent an ECG signal. However, the algorithm needed to be evaluated with more patients to determine the range of parameters in which a healthy ECG signal can be generated by our model. Therefore, we ran the algorithm for each of the 90 patients in the database, and the same procedure as shown in Algorithm 1 was executed, where a set of random solutions were proposed for each patient. Subsequently, the performance of each random solution was evaluated with Equation (14). The best solutions had a crossover and mutation stage, and, finally, a new selection of the best solutions was made. This procedure was repeated until the number of proposed iterations in Table 1 was completed. Once the algorithm had been executed for a heartbeat per patient, a set of 90 vectors were obtained with coefficients that fit each patient’s ECG with the model wave. The mean in each parameter and their standard deviation were registered in Table 3. Additionally, we calculated statistical indicators for the 90 patients, obtaining the following average values: RMSE of 0.0543, $R^{2}$ of 0.6913, and MBE of −0.0407.

**Figure 4:**
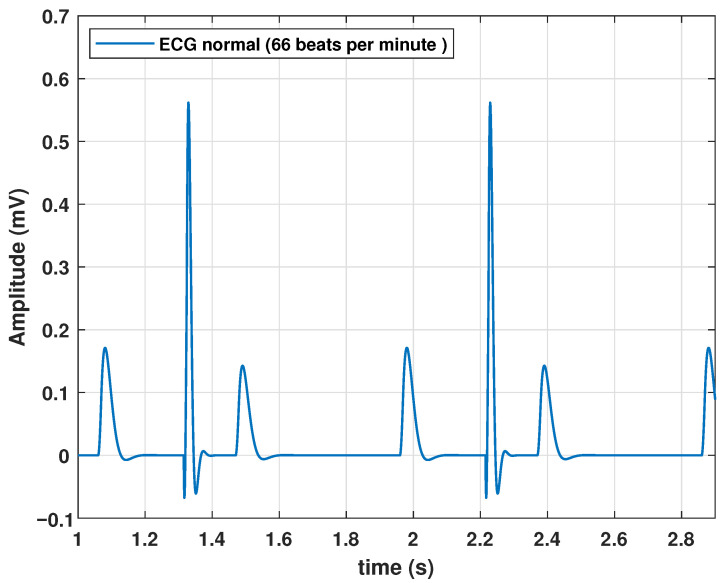
Response of the proposed model to a periodic impulse input.

Although the algorithm performed correctly and the model could generate a heartbeat, a series of quasi-cyclic heartbeats determined the periodic nature of the ECG signal. Therefore, we added a frequency parameter *f* to (13). The normal frequency of a healthy adult at rest is 60–90 beats per minute (bpm). Therefore, for the parameters listed in Table 2, a frequency of 1.11 Hz was added. This parameter value resulted in a cardiac frequency of 66 bpm. The periodic behavior of the ECG with the model based on the transfer functions is shown in Figure 4.

Notably, the natural frequency of the model was regulated by a single parameter, thereby simplifying the adjustment of the heart rate. The primary goal of this study was to ensure that the model effectively controlled the signal dynamics, producing ECGs representative of common heart conditions. To achieve this, four typical heart problems were modeled: sinus tachycardia, atrial flutter, atrial flutter, and ventricular Flutter.

**Figure 5:**
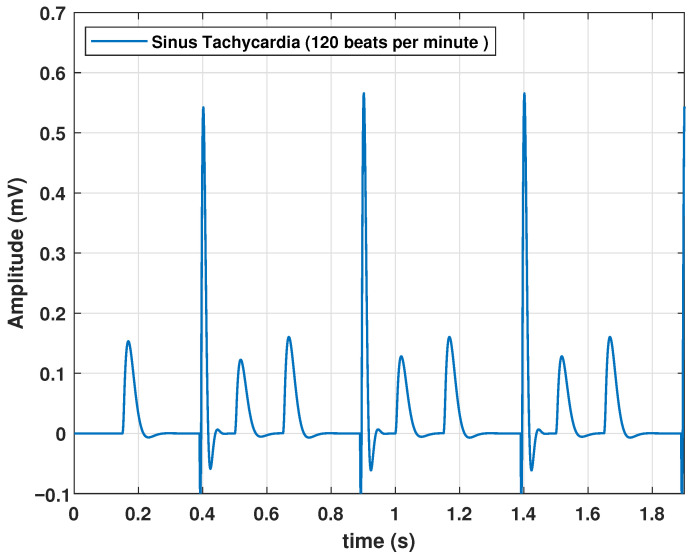
Synus Tachycardia signal obtained with the proposed model, with an input frequency of 2 Hz.

Sinus tachycardia is an elevation in heart rate, even when an individual is at rest. Although the normal value depends on factors such as age and physical activity, a value greater than 100 bpm in healthy adults at rest is indicative of tachycardia. Therefore, a higher frequency is required for this type of pathology. To achieve this, we configured *f* at 2 Hz, resulting in a heart rate of 120 bpm, as shown in Figure 5.

The other arrhythmias considered in this work imply changes in the waveform, so the system parameters were readjusted to reproduce them. By employing the same methodology based on a GA, we determined the correct combination of parameters to produce ECG waveforms resembling cardiac arrhythmias. For this purpose, the public database MIT-BIH Arrhythmia Database was used, which is a set of more than 4000 long-term Holter records that were obtained by the Arrhythmia Laboratory of Beth Hospital [32,33].

This database contains 48 records with a duration of 30 min, corresponding to 25 men between 32 and 89 years old and 22 women between 23 and 89 years old. These records were selected to include a variety of rare but clinically essential phenomena that a small random sample would not represent well.

Signals were bandpass-filtered and digitized at 360 Hz using hardware built at the MIT Biomedical Engineering Center and BIH Biomedical Engineering Laboratory. The signals had a resolution of 11 bits in the range of ±5 mV. Therefore, the sample values ranged between 0 and 2047, where zero volts correspond to 1024.

**Figure 6:**
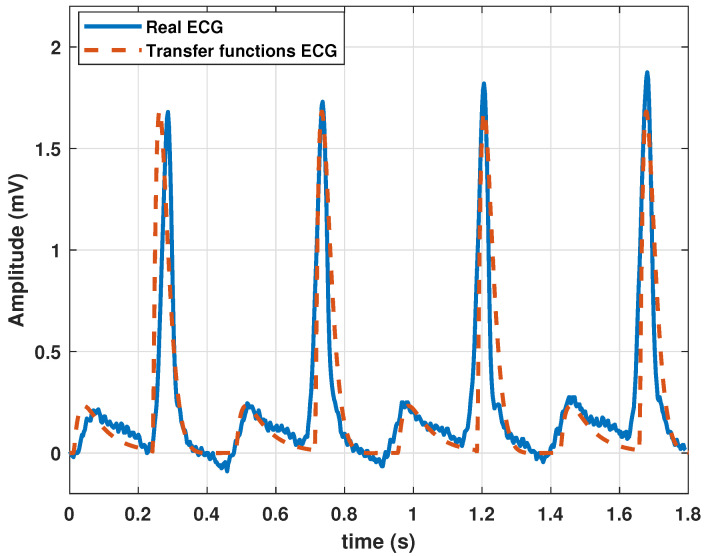
Atrial flutter signal obtained with the proposed model with parameter values given in Table 4.

After proper parameter adjustment, our model could reproduce atrial flutter, which indicates an abnormal heart rhythm that begins in the atrial chambers of the heart. It is generally associated with a rapid heart rate and is classified as supraventricular tachycardia. Atrial flutter is characterized by a frequency between 220 and 350 bpm. The rhythm can be regular, but P waves do not appear and are replaced by flutter (F) waves, which are more similar to sawtooth or triangular waves. A complex QRS usually lasts no more than 0.12 s. This type of pathology could be reproduced by the proposed model with the parameters listed in Table 4, and the resulting wave is shown in Figure 6.

**Figure 7:**
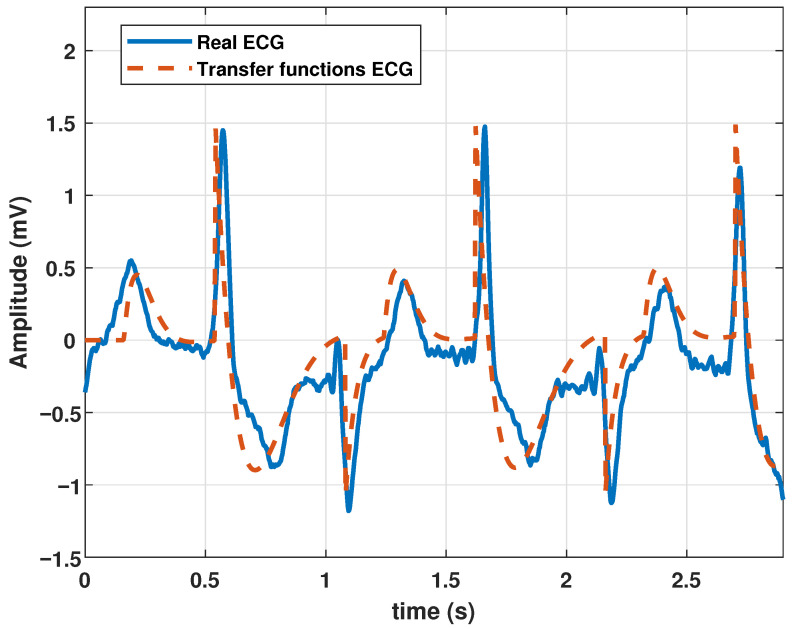
Ventricular tachycardia signal obtained with the proposed model with parameter values given in Table 5.

Additionally, our model can generate ventricular tachycardia, which is characterized by atypical electrical signals in the ventricles, which can last a few seconds or longer. When ventricular tachycardia occurs, the heart beats faster than 100 bpm. A disorderly heartbeat prevents the chambers of the heart from adequately filling with blood. Consequently, the heart may be incapable of pumping sufficient blood. The parameters listed in Table 5 were used to simulate this arrhythmia. The resulting ECG is shown in Figure 7.

**Figure 8:**
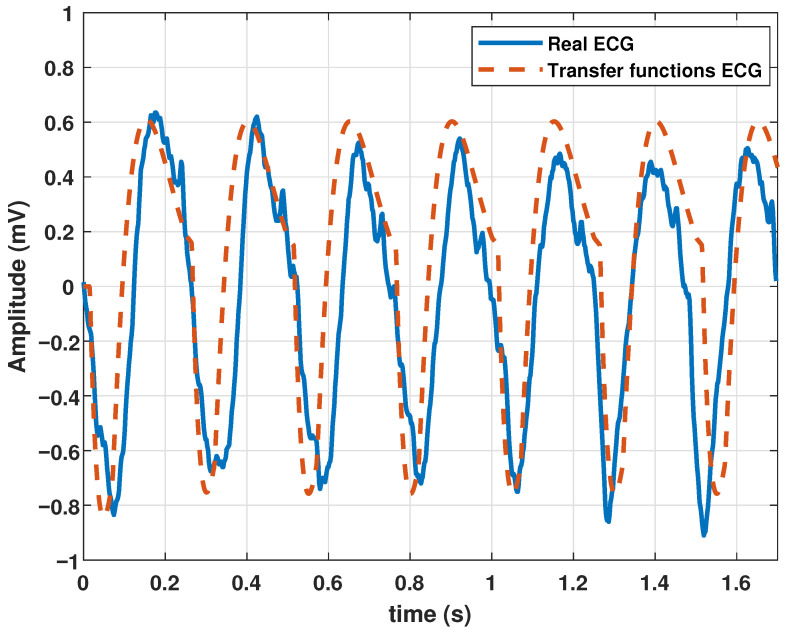
Ventricular flutter signal obtained with the proposed model with the parameters given in Table 6.

The model was also tested to reproduce ventricular flutter waveforms. Ventricular flutter is a severe ventricular arrhythmia (originates in the lower chambers of the heart or ventricles). It manifests as rapid heart contractions exceeding 200 bpm. In the ECG recordings, a continuous sinusoidal pattern devoid of QRS complexes or T waves emerges. Utilizing the parameter values in Table 6, the model generated a ventricular flutter signal, as depicted in Figure 8.

Thus far, we have shown that the model is capable of representing heartbeats, healthy electrocardiogram signals, and ECG signals for different diseased hearts.

Finally, it was adequate to compare the proposed model with other models with the same aim of generating ECG signals. This comparison is presented in Table 7.

From the comparison given in Table 7, we can conclude that the proposed model is linear and has a number of parameters similar to the model based on quasiperiodic motion [25], but the main advantage is the control of variables with parameter variations, which are determined by means of a GA, and no manual tuning is required. Each model may have advantages or disadvantages depending on the application and precision required.

## 5. Conclusions

In this study, we presented a mathematical model based on transfer functions to reproduce the electrical activity of the heart at the macroscopic level through ECG signals. Our model comprises a set of three transfer functions coupled with time delays with parameters adjusted via a GA. By fine-tuning the parameters with clinical ECG records as reference signals, we generated waveforms that resembled real ECG registers, with a root mean square error of 4.7% and an $R^{2}$ value of 0.72. The simplicity of our model allows for easy adjustment of the waveform parameters, enabling the generation of both normal and arrhythmic signals. Despite its linear nature, the ability of our model to reproduce specific heartbeats makes it a valuable tool for assessing and testing ECG-monitoring equipment.

Compared to existing models, our approach closely aligns with periodically kicked RLC oscillator models, but with a simpler trigonometric function-based approach. Unlike the manual adjustment in most of the existing models, our method offers a systematic approach for automatically tuning parameters through a GA. However, it is worth mentioning that the linear representation of the model makes it impossible to capture the nonlinear complexities of cardiac electrical activity. Nonetheless, the structured nature of our transfer functions holds potential for future arrhythmia control initiatives.

In future work, generalization to include multi-lead ECGs and the representation of bundle branch blocks and ischemia, such as ST elevation, present exciting avenues for research. Additionally, exploring ECG classification through parametric estimation using AI-based multidimensional classifiers offers further opportunities for advancement in this field.

## Figures and Tables

**Table 1:** Parameter values of the Genetic Algorithm.

GA Hyperparameters	Value	Details
Population	5000	Numbers of vectors with random coefficients
Upper search limit	[300 300 80,000 80,000 300 0.2 0.6 0.8 5]	Maximum values allowed for parameters $a_{i},b_{i},c_{i},d_{i},k_{i},r_{1},r_{2},r_{3},$ and *f*, respectively (parameters with subscript *i* indicate that they share the same limit for the 3 values of *i*).
Lower search limit	[−300 0 0 0 −300 0.001 0.2 0.4 1]	Minimum values allowed for parameters $a_{i},b_{i},c_{i},d_{i},k_{i},r_{1},r_{2},r_{3},$ and *f*, respectively
Generations	6	Stop condition
Fitness function	$ISE=\int \varepsilon^{2}dt$	Function for evaluate the performance of each individual (integral square error)
Elitism	10%	Numbers of individuals in the search
Biological pressure	70%	Percentage of individuals that reproduce
Mutation probability	30%	Probability of random mutation occurs

**Table 2:** Results of the genetic algorithm for estimating the coefficients in a heartbeat.

Parameter	i = 1	i = 2	i = 3
r	0.0794	0.192	0.28
a	0	−0.465	0
b	40.92	349.96	35.12
c	120.22	223.35	38.90
d	0.85 × $10^{3}$	48.3 × $10^{3}$	2.28 × $10^{3}$
k	50.11	94.81	57.57

**Table 3:** Range of parameter values in transfer function model for healthy ECG signals.

Parameter	i = 1		i = 2		i = 3	
	mean	SD	mean	SD	mean	SD
r	0.0801	0.0395	0.28	0.32	0.43	0.27
a	0.093	0.19	−0.53	0.27	0.076	0.08
b	42.87	19.76	310.49	62.11	24.58	12.56
c	106.65	31.21	218.77	22.65	55.43	44.45
d	3.5 × $10^{3}$	5.8 × $10^{3}$	35.1 × $10^{3}$	25.2 × $10^{3}$	6.7 × $10^{3}$	8.4 $\times \;10^{3}$
k	51.29	44.77	88.9	31.5	44.9	21.5

**Table 4:** Coefficients in the transfer function Model (13) used to generate an atrial flutter signal.

Parameter	i = 1	i = 2	i = 3
r	0.0001	0.23	0.34
a	−0.03	−0.1	−0.006
b	49.84	501.04	58.34
c	79.81	100.56	44.33
d	1.02 × $10^{3}$	2.98 × $10^{3}$	0.5 × $10^{3}$
f	2.118		
k	99.98	101.34	0.009

**Table 5:** Coefficients of the proposed model to generate a ventricular tachycardia signal.

Parameter	i = 1	i = 2	i = 3
r	0.15	0.53	1.07
a	0.004	−14.23	−5.8
b	119.82	148.36	0.007
c	31.5	14.65	22.41
d	401.12	102.34	10.65
f	0.92		
k	80.01	49.80	89.78

**Table 6:** Coefficients of the proposed model to generate a ventricular tachycardia signal.

Parameter	i = 1	i = 2	i = 3
r	0.00095	0.068	0.2
a	0	0	0
b	10.2	9.78	10.02
c	23.02	22.91	22.97
d	300.12	299.88	300.09
f	4.01		
k	500.11	599.11	149.34

**Table 7:** Comparison of the proposed model with some other models with similar purposes.

Model	Reference	Number of Equations	Number of Parameters	Linearity	Parameter Adjustment
**Proposed here**	-	3 (transfer functions)	19	Yes	CGA
**Discretized reaction–diffusion**	[24]	4 (differential equations)	9	No	Manual
**Heterogeneous nonlinear oscillators**	[22]	4 (differential equations)	48	No	Manual
**Ring of three coupled oscillators**	[21]	6 (differential equations)	34	No	Manual
**Model based on a quasiperiodic motion**	[25]	5 (differential equations)	19	No	Manual
**Periodically kicked network of RLC oscillators**	[27]	4 (differential equations)	8	Yes	Manual

## Data Availability

The database for healthy ECGs (ECG-ID database) is available at https://physionet.org/content/ecgiddb/1.0.0/ (accessed on 1 April 2024) and MIT-BIH Arrhythmia Database is available at: https://www.physionet.org/content/mitdb/1.0.0/ (accessed on 1 April 2024).

## References

[1] Motta S., Pappalardo F. (2012). Mathematical modeling of biological systems. Briefings Bioinform..

[2] Michail M., Brown A.J. (2018). Physiology of the normal heart. Medicine.

[3] Silvani A., Calandra-Buonaura G., Dampney R.A.L., Cortelli P. (2016). Brain–heart interactions: Physiology and clinical implications. Philos. Trans. R. Soc. A Math. Phys. Eng. Sci..

[4] Nowbar A.N., Gitto M., Howard J.P., Francis D.P., Al-Lamee R. (2019). Mortality From Ischemic Heart Disease. Circ. Cardiovasc. Qual Outcomes.

[5] Kaplan Berkaya S., Uysal A.K., Sora Gunal E., Ergin S., Gunal S., Gulmezoglu M.B. (2018). A survey on ECG analysis. Biomed. Signal Process. Control..

[6] Keener J., Sneyd J. (2009). The Heart. Mathematical Physiology: II: Systems Physiology.

[7] Liu X., Wang H., Li Z., Qin L. (2021). Deep learning in ECG diagnosis: A review. Knowl.-Based Syst..

[8] Houssein E.H., Kilany M., Hassanien A.E. (2017). ECG signals classification: A review. Int. J. Intell. Eng. Inform..

[9] Prusty M.R., Pandey T.N., Lekha P.S., Lellapalli G., Gupta A. (2024). Scalar invariant transform based deep learning framework for detecting heart failures using ECG signals. Sci. Rep..

[10] Alonso S., dos Santos R.W. (2019). Modelling the Electrical Activity of the Heart. Cardiovascular Computing—Methodologies and Clinical Applications.

[11] Peirlinck M., Costabal F.S., Yao J., Guccione J.M., Tripathy S., Wang Y., Ozturk D., Segars P., Morrison T.M., Levine S. (2021). Precision medicine in human heart modeling. Biomech. Model. Mechanobiol..

[12] Jæger K.H., Tveito A. (2022). Deriving the Bidomain Model of Cardiac Electrophysiology from a Cell-Based Model; Properties and Comparisons. Front. Physiol..

[13] Trudel M.C., Dube B., Potse M., Gulrajani R., Leon L. (2004). Simulation of QRST integral maps with a membrane-based computer heart model employing parallel processing. IEEE Trans. Biomed. Eng..

[14] Barrio R.A., Dominguez-Roman I., Quiroz-Juarez M.A., Jimenez-Ramirez O., Vazquez-Medina R., Aragon J.L. (2019). Modelling the electrical activity of the heart. Mathematical Biology and Biological Physics.

[15] Quiroz-Juárez M.A., Rosales-Juárez J.A., Jiménez-Ramírez O., Vázquez-Medina R., Aragón J.L. (2022). ECG Patient Simulator Based on Mathematical Models. Sensors.

[16] Montoya-Santiyanes L.A., Chay-Canul A.J., Camacho-Pérez E., Rodríguez-Abreo O. (2022). A novel model for estimating the body weight of Pelibuey sheep through Gray Wolf Optimizer algorithm. J. Appl. Anim. Res..

[17] Ogata K. (1999). Modern control engineering. Book Rev..

[18] Kuo B.C. (1987). Automatic Control Systems.

[19] Hampton J., Hampton J. (2019). The ECG Made Easy E-Book.

[20] Bueno-Orovio A., Britton O., Muszkiewicz A., Rodriguez B., Bradshaw R.A., Stahl P.D. (2016). Cardiac Modeling. Encyclopedia of Cell Biology.

[21] Gois S.R., Savi M.A. (2009). An analysis of heart rhythm dynamics using a three-coupled oscillator model. Chaos Solitons Fractals.

[22] Ryzhii E., Ryzhii M. (2014). A heterogeneous coupled oscillator model for simulation of ECG signals. Comput. Methods Programs Biomed..

[23] Quiroz-Juárez M., Vázquez-Medina R., Ryzhii E., Ryzhii M., Aragón J. (2017). Quasiperiodicity route to chaos in cardiac conduction model. Commun. Nonlinear Sci. Numer. Simul..

[24] Quiroz-Juárez M.A., Jiménez-Ramírez O., Vázquez-Medina R., Breña-Medina V., Aragón J.L., Barrio R.A. (2019). Generation of ECG signals from a reaction-diffusion model spatially discretized. Sci. Rep..

[25] McSharry P., Clifford G., Tarassenko L., Smith L. (2003). A dynamical model for generating synthetic electrocardiogram signals. IEEE Trans. Biomed. Eng..

[26] Sayadi O., Shamsollahi M.B., Clifford G.D. (2010). Synthetic ECG generation and Bayesian filtering using a Gaussian wave-based dynamical model. Physiol. Meas..

[27] Quiroz-Juárez M., Jiménez-Ramírez O., Aragón J., Del Río-Correa J., Vázquez-Medina R. (2019). Periodically kicked network of RLC oscillators to produce ECG signals. Comput. Biol. Med..

[28] Taff L. (1991). Expanding the scientific role of the hubble space telescope fine guidance sensors. Adv. Space Res..

[29] Agrawal P., Abutarboush H.F., Ganesh T., Mohamed A.W. (2021). Metaheuristic Algorithms on Feature Selection: A Survey of One Decade of Research (2009–2019). IEEE Access.

[30] Bhoskar M.T., Kulkarni M.O.K., Kulkarni M.N.K., Patekar M.S.L., Kakandikar G., Nandedkar V. (2015). Genetic Algorithm and its Applications to Mechanical Engineering: A Review. Mater. Today Proc..

[31] Lugovaya T.S. (2005). Biometric Human Identification Based on Electrocardiogram. Ph.D. Thesis.

[32] Goldberger A., Amaral L., Glass L., Hausdorff J., Ivanov P., Mark R., Stanley H., PhysioBank P. (2000). PhysioNet: PhysioBank, PhysioToolkit, and PhysioNet: Components of a new research resource for complex physiologic signals Components of a new research resource for complex physiologic signals. Circulation.

[33] Moody G., Mark R. (2001). The impact of the MIT-BIH Arrhythmia Database. IEEE Eng. Med. Biol. Mag..

